# Posttranslational Modifications of Red Blood Cell Ghost Proteins as “Signatures” for Distinguishing between Low- and High-Risk Myelodysplastic Syndrome Patients

**DOI:** 10.4274/tjh.2016.0251

**Published:** 2017-03-01

**Authors:** Klara Pecankova, Pavel Majek, Jaroslav Cermak, Jan E. Dyr

**Affiliations:** 1 Institute of Hematology and Blood Transfusion, Prague, Czech Republic

**Keywords:** Myelodysplastic syndromes, proteomics, Red blood cell ghosts, Posttranslational modifications, Erythrocytes, Electrophoresis

## TO THE EDITOR,

Myelodysplastic syndrome (MDS) comprises a heterogenic group of oncohematological diseases that affect hematopoiesis. Although the precise cause of MDS is unknown, multiple factors are involved, one of the most widely implicated of which is oxidative stress. However, it is unclear whether oxidative stress is a cause of MDS or an effect of other pathological mechanisms.

Red blood cells (RBCs) are the first cells exposed to stress stimuli. They are highly vulnerable to free radical accumulation, which leads to the oxidative stress that induces damage in proteins and other biomacromolecules [1]. In MDS, the RBC proteome can be affected by effects of the peripheral blood environment and/or by abnormal processes possibly caused by oxidative stress during hematopoiesis in bone marrow. Therefore, we chose red cell membranes (ghosts) as a model biological material.

Patient characteristics are summarized in Table 1. All individuals tested agreed to participate in the study on the basis of informed consent. All samples were obtained and analyzed in accordance with the Ethics Committee regulations of the Institute of Hematology and Blood Transfusion. The RBCs were isolated from whole blood by differential centrifugation and frozen at -80 °C. The red cell ghosts were isolated according to the method of Dodge et al. [2]. Proteins were separated using 2D SDS-PAGE followed by silver staining [3]. The gels were digitized and processed using Progenesis SameSpots software. Significantly differing spots (p<0.05) were submitted for protein identification by tandem mass spectrometry coupled to a Nano LC system.

By comparing the high- and low-risk MDS ghost proteomes, we found 22 significantly differing spots that corresponded to 16 unique proteins, particularly spectrin and its interaction partners in the membrane skeleton meshwork (actin, tropomodulin, tropomyosin, ankyrin, protein 4.1). To determine whether the changes were caused by protein expression level alterations or by posttranslational modifications, we analyzed the LC-MS data using Progenesis LC-MS software. No significant changes in protein expression levels were observed. Therefore, the changes between the low- and high-risk MDS cohorts were caused by protein modifications.

It is well known that RBC function may be radically affected by membrane protein posttranslational modifications. For example, the increased phosphorylation of spectrin, the protein crucial for cytoskeletal stability, worsens the mechanical properties of the RBC membrane [4], and the phosphorylation of protein 4.1 leads to RBC osmotic fragility. Moreover, increased protein 4.1 mobility, suggesting the presence of other modifications, has been observed in MDS patients. The production of abnormal protein 4.1 may result in the dysregulation of spectrin-actin interaction and may cause both RBC shape change and membrane instability [5]. In addition, altered membrane morphology, including holes and thorn-like structures, was described for the RBCs of MDS and acute myeloid leukemia patients [6].

Because the impact of RBC protein modifications is clear, our preliminary data suggest that it might be possible to profile MDS patients according to the type of modification. Such modifications could thus be considered characteristic signatures of MDS or its progression. As such, they might help in the earlier diagnosis and treatment of MDS patients.

## Figures and Tables

**Table 1:**
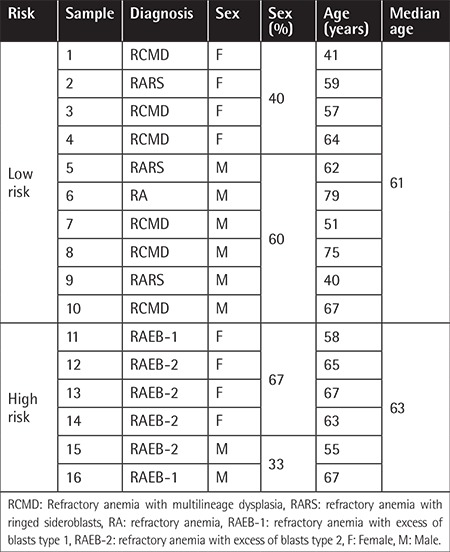
Patient characteristics.
